# HSD10 disease in a female: A case report and review of literature

**DOI:** 10.1002/jmd2.12250

**Published:** 2021-09-15

**Authors:** Jariya Upadia, Nicolette Walano, Grace S. Noh, Jiao Liu, Yuwen Li, Stephen Deputy, Lindsay T. Elliott, Joaquin Wong, Jennifer A. Lee, Raymond C. Caylor, Hans C. Andersson

**Affiliations:** ^1^ Hayward Genetics Center, Department of Pediatrics Tulane University School of Medicine New Orleans Louisiana USA; ^2^ Department of Pediatrics Tulane University School of Medicine New Orleans Louisiana USA; ^3^ Division of Pediatric Neurology, Department of Pediatrics Louisiana State University Health Sciences Center/Children's Hospital New Orleans Louisiana USA; ^4^ Department of Pediatric Physical Medicine and Rehabilitation Louisiana State University Health Sciences Center/Children's Hospital New Orleans Louisiana USA; ^5^ Greenwood Genetic Center Greenwood South Carolina USA

**Keywords:** 2‐methyl‐3‐hydroxy‐butyryl‐CoA dehydrogenase (MHBD) deficiency, HSD10 disease, mitochondrial disorder, skewed X inactivation, X‐chromosome inactivation (XCI), X‐chromosome inactivation study

## Abstract

HSD10 disease is a rare X‐linked mitochondrial disorder caused by pathogenic variants in the *HSD17B10* gene. The phenotype results from impaired 17β‐hydroxysteroid dehydrogenase 10 (17β‐HSD10) protein structure and function. HSD10 is a multifunctional protein involved in enzymatic degradation of isoleucine and branched‐chain fatty acids, the metabolism of sex hormones and neurosteroids, as well as in regulating mitochondrial RNA maturation. HSD10 disease is characterised by progressive neurologic impairment. Disease onset is varied and includes neonatal‐onset, infantile‐onset and late‐onset in males. Females can also be affected. Our index case is a 45‐month‐old female, who initially presented at 11 months of age with global developmental delay. She subsequently began to lose previously acquired cognitive and motor skills starting around 29 months of age. Brain MRI showed abnormalities in the basal ganglia indicative of possible mitochondrial disease. Urine organic acid analysis revealed elevations of 2‐methyl‐3‐hydroxybutyric acid and tiglyglycine. *HSD17B10* gene sequencing revealed a likely pathogenic variant, NM_001037811.2:c.439C>T (p.Arg147Cys) inherited from her mother, expected to be causative of HSD10 disease. Her X‐chromosome inactivation study is consistent with a skewed X‐inactivation pattern. We report a female patient with HSD10 disease caused by a missense pathogenic variant, Arg147Cys in the *HSD17B10* gene. The patient is the fifth severely affected female with this disease. This case adds to the small number of known affected families with this highly variable disease in the literature. These findings support the possibility of X‐inactivation patterns influencing the penetrance of HSD10 disease in females.

**Synopsis**

HSD10 disease is a rare X‐linked mitochondrial disorder, a hallmark feature of this disorder is impairment of HSD10 protein function. 17β‐HSD10 is a multifunctional protein involved in various metabolic pathways as well as mitochondrial tRNA processing. Basic urine organic acid analysis typically reveals elevated levels of intermediate metabolites of isoleucine metabolism. Most affected individuals have neurological regression in the early infantile or early childhood period. Approximately 13% of females are severely affected. Data suggests that phenotypic severity among females may be linked to X‐chromosome inactivation (XCI).

## 1 INTRODUCTION

17β‐Hydroxysteroid dehydrogenase type 10 (17β‐HSD10) deficiency or HSD10 mitochondrial disease (OMIM 300256) is an X‐linked disorder caused by dysfunction of the 17β‐HSD10 protein. 17β‐HSD10 is encoded by the *HSD17B10* gene, which maps to chromosome Xp11.2. The 17β‐HSD10 protein is a multifunctional enzyme, involved in the metabolism of isoleucine^1^ and branched chain fatty acids.^2^ This protein is a component of mitochondrial ribonuclease P (RNase P) localised in mitochondria,^3^ which contributes to mitochondrial RNA posttranscriptional processing.^4^, ^5^ Moreover, 17β‐HSD10 protein also plays an important role in the metabolism of sex steroid hormones, neurosteroids,^6^ and the maintenance of neurosteroid homeostasis; all critical to brain development.^7^ Pathology of HSD10 disease is thought to be caused by general mitochondrial dysfunction owing to its role in mitochondrial tRNA processing^4^, ^8^ along with abnormal neurosteroidogenesis.^7^ 17β‐HSD10 protein is found in various human tissues and is most abundant in liver.^3^ Levels of 17β‐HSD10 protein vary among different brain regions, it is found most abundantly in the hippocampus. The hypothalamus, thalamus, hippocampus, and brain stem have higher 17β‐HSD10 protein levels compared to the cerebral cortex, cerebellum and spinal cord.^9^ Pathogenic variants in the *HSD17B10* gene cause HSD10 disease, which is also known as 2‐methyl‐3‐hydroxybutyryl‐CoA dehydrogenase (MHBD) deficiency due to the enzymes activity in the isoleucine degradation pathway, resulting in elevation of 2‐methyl‐3‐hydroxybutyrate and tiglylglycine in urine organic acid analysis.^10^, ^11^ This older nomenclature is felt to be misrepresentative of the complex pathophysiology of this disease therefore, HSD10 disease is the preferred nomenclature.

HSD10 disease is a rare X‐linked disorder characterised by progressive neurodegeneration, seizures, cardiomyopathy, hypotonia, ataxia, microcephaly, and visual impairment. This disease is most commonly described in males, but females may also be affected due to non‐random X‐chromosome inactivation (XCI).^12^, ^13^ The onset of this disease may be neonatal, infantile or late childhood onset. The phenotypic spectrum ranges from asymptomatic to severe with progressive neurological regression in the neonatal period.^12^, ^14^, ^15^, ^16^ Severely affected children may die in infancy or early childhood as a result of fulminant cardiac failure, refractory lactic acidosis or neurological regression.^12^, ^14^, ^15^ A few males with no or minimal neurological involvement have been reported.^16^, ^17^, ^18^, ^19^ The neurologic examination can reveal microcephaly, truncal hypotonia, ataxia, myoclonus, choreoathetoid movements, dystonia, and spasticity.^15^, ^16^, ^20^, ^21^, ^22^ Ophthalmologic manifestation includes nystagmus, astigmatism, strabismus, optic atrophy, retinopathy, pigmentary retinopathy, and non‐pigmentary retinopathy.^15^, ^20^, ^22^ Patients may present during the first few days of life with lactic and metabolic acidosis, as well as hypotonia.^12^, ^20^ Regression and/or metabolic decompensation may be triggered by illness.^14^, ^21^ The diagnosis of HSD10 disease is established in affected individuals with elevation of 2‐methyl‐3‐hydroxybutyrate and tiglylglycine in urine organic acid analysis plus the detection of a *HSD17B10* pathogenic variant or significantly reduced activity of the MHBD enzyme in fibroblasts or leukocytes. It should be mentioned that the level of enzyme activity does not correlate well with disease severity.^15^, ^19^ HSD17B10 pathogenic variants disrupt the structure and function of mitochondrial RNase P protein complex and this disruption is independent of MHBD enzyme activity.^4^, ^23^ Acylcarnitine profile can be normal or slightly abnormal with mild elevation of C5:1 with or without elevation of C5‐OH.^10^, ^20^, ^24^ There is currently no effective treatment of clinical symptoms. Dietary limitation of isoleucine intake has been shown to reduce 2‐methyl‐3‐hydroxybutyrate and tiglylglycine in urine, however this has been reported without clinical improvement.^15^ Brain imaging abnormalities in individuals with HSD10 disease are variable and may include frontotemporal atrophy, diffuse cerebral atrophy, decreased white matter volume, progressive cortical and subcortical atrophy, periventricular white matter lesions, hypoxic ischemic encephalopathy (HIE)‐like lesions, enlarged ventricles, occipital infarction, signal abnormality in putamen, increased T2 signal in the basal ganglia, dentate nuclei and pons, and Leigh‐like lesions.^11^, ^12^, ^22^, ^24^, ^25^ Normal MRI findings have been reported in some cases.^12^, ^16^ An elevation of brain lactate can be seen by MR spectroscopy in some cases.^8^, ^12^

Since its first description in 2000,^20^ 34 index cases and 29 relatives from different ethnic backgrounds with pathogenic variants in *HSD17B10* gene have been reported. Approximately 10% of index cases were females. Among 4 index females, 3 cases were reported to have infantile onset neurological features,^12^, ^24^, ^26^ and 1 case was reported to have a pathogenic variant in CACNA1A gene in addition to HSD10 disease which could have synergistically contributed to her neurologic symptoms.^16^ It was previously reported that this gene is the first of the gene cluster at Xp11.2 to escape inactivation.^27^ Approximately 10% of X‐linked genes show varied degrees of expression between female individuals and tissues, which results in a range of disease severity in heterozygous females.^28^, ^29^ However, this conclusion is not consistent between studies. A subsequent quantification study of *HSD17B10* cDNA in skin fibroblasts indicated that this gene does not escape XCI. This may point to potential effects of XCI in the severity of HSD10 disease in females.^13^ Skewed XCI or non‐random XCI is generally known to play a role in the clinical variability of X‐linked disorders seen in female carriers of X‐linked dominant conditions. Skewed XCI is common in females and the extent of skewed XCI differs greatly.^30^

In this report, we describe an African American female with X‐linked HSD10 disease caused by an NM_001037811.2:c.439C>T (p.Arg147Cys) pathogenic variant identified by *HSD17B10* gene sequencing who presented with neurologic deterioration and an abnormal brain MRI. In this study, we present a severely affected female with skewed XCI, and explore the role of XCI in modulating HSD10 disease in females.

## 2 CASE PRESENTATION

**FIGURE 1:**
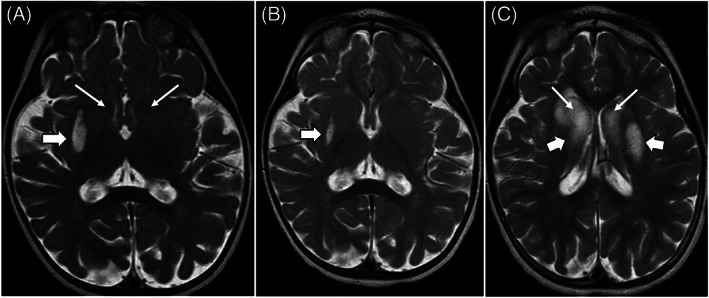
(A) T2 axial MRI of the Brain from 11 months of age. An edematous lesion is present within the right putamen nucleus (large arrow). The caudate nuclei remain radiographically unaffected (thin arrows). (B) T2 axial MRI of the brain at 15 months of age showing the formerly edematous right‐sided lesion of the putamen evolving into cystic cavitation (thick arrow). (C) T2 axial MRI of the brain at 2 years, 5 months of age showing massive edematous changes of the caudate nuclei (thin arrows), as well as of the putamen nuclei bilaterally (thick arrows)

We report a 45‐month‐old African American female who was admitted to our facility for evaluation of developmental delay and regression. She was born at 34‐weeks gestational age via C‐section to a 28‐year‐old G2P1 mother. Post‐natal course was complicated by mild jaundice and bradycardia, which resolved before discharge. Newborn screen by tandem mass spectrometry was normal. Family history is positive for mild learning disability in her mother. At 11 months of age, she was noted to have global developmental delay and low muscle tone; she was not able to sit or crawl. The patient started to sit independently by age 17 months and walk by age 2 years. At 18 months of age, she was referred for genetic evaluation. Chromosome SNP microarray analysis, plasma amino acid analysis and urine organic acid analysis were reported to be normal. A series of MRI scans of the brain have evolved over time. Her first brain MRI at age 11 months shows an isolated edematous lesion affecting the lateral aspect of the right putamen nucleus (Figure 1A). A follow‐up MRI of the brain at age 15 months shows evolution of the right putamen lesion into cystic cavitation (Figure 1B).

At 29 months of age, she experienced her first seizure, described as a tonic‐clonic seizure, and began to show regression in her cognitive and motor functions. Her most recent brain MRI at age 31 months shows significant confluent edematous changes in both caudate nuclei as well as scattered edematous changes within the putamen nuclei bilaterally with sparing of the thalamus, globus pallidus and brainstem nuclei (Figure 1C). At age 32 months, she was admitted for further investigation and treatment. At her peak abilities, she was able to walk and run, feed herself with her hands and say approximately two words. On admission, she was not able to walk, stand or feed herself. Examination revealed normal growth parameters: weight 12.1 kg (26.5th centile), height 86 cm (4th centile), and head circumference 45.5 cm (5th centile). She had no evidence of dysmorphic features or craniofacial anomalies. Her neurologic examination was notable for low muscle tone on all extremities. Deep tendon reflexes were 2+ in the biceps and patella bilaterally without clonus. Ophthalmologic examination showed vascular attenuations, pale optic disc and retinal dystrophy of both eyes. Auditory brainstem response was reported to be normal. Echocardiogram showed normal cardiac anatomy and function. Electrocardiogram was reported to be normal. Blood lactate was mildly elevated at 2.9 mmol/L (0.5‐2.2 mmol/L), urine organic acid analysis showed an increase in lactic acid, 2‐methyl‐3‐hydroxybutyric acid and tiglyglycine without elevation of 2‐methyacetoacetic acid, which is consistent with 2‐methyl‐3‐hydroxybutyryl‐CoA Dehydrogenase deficiency (MHBDD, or HSD10 disease). *HSD17B10* gene sequencing revealed a likely pathogenic variant, NM_001037811.2:c.439C>T (p.Arg147Cys). In silico analysis support that this variant has a deleterious effect on protein structure and function.^31^ Her clinical presentation, biochemical findings and molecular result were consistent with the diagnosis of HSD10 disease. The patient has inherited this variant from her mother. To elucidate the factors contributing to distinct clinical phenotypes in the proband and her mother, XCI analysis of the Androgen Receptor (AR) locus was performed on genomic DNA from blood samples from the patient and her mother. XCI ratios of 77:23 and 68:32 were observed in the patient and her mother, respectively.

On most recent follow‐up visit to assess functional status at age 37 months, the patient's mother reported increased mobility. She was non‐verbal. She was able to take a few independent steps without support and has frequent falls with a widened base of support and over‐pronation of the feet. Caregivers reported that she could self‐feed with her hands, but she had difficulty manipulating and effectively grasping a utensil to bring food to her mouth.

## 3 DISCUSSION

We report an Arg147Cys variant in *HSD17B10* gene in an African American female patient with HSD10 disease. HSD10 disease is a rare X‐linked mitochondrial disorder, which occurs primarily in males and rarely in females. Thus far, only four females have been reported to have neurologic features related to HSD10 disease.^12^, ^16^, ^24^, ^26^ However, one case had co‐occurring disease which very likely contributed to her neurologic symptoms.^16^ Several female carriers have not shown signs and symptoms of neurologic regression. There have been 16 different missense variants reported to cause HSD10 disease.^12^, ^15^, ^16^, ^17^, ^22^, ^26^, ^32^, ^33^ Our case also has a missense variant that has not been previously described in literature. A correlation between the *HSD17B10* variants and clinical severity has not been established in the majority of cases due to the rarity of each variant. Arg130Cys is the most frequent variant seen in approximately 30% of index cases.

The *HSD17B10* gene has 261 codons in its main transcript NM_004493.3. Referring to the functional domain map for *HSD17B10*‐encoded HSD10 protein,^8^ the N‐terminus of the HSD10 protein contains the NAD‐binding domain (codons 17‐23) and subunit interaction domains (covered within codons 100‐146), while the C‐terminus contains the dehydrogenase domain (covered within codons 155‐172), the substrate binding domain (codons 203‐220), and the tetramerization domain (in the remaining coding region). As previously described, the dehydrogenase activity was not a reliable indicator for predicting the phenotypes. The protein structural changes in the functional tetramer complex and the successive functional damages to the tetramer may contribute more to the disease severity. A potential correlation between disease severity and the mutated domain was observed. Pathogenic variants around the dehydrogenase domain (Ala154Thr, Ala157Val, and Gln165His) contributed to the non‐progressive/mild disease type, while those around the subunit interaction domain (Asp86Gly, Leu122Val, Arg130Cys, and the present variant Arg147Cys), the substrate binding domain (Arg192Arg=, Pro210Ser, and Lys212Glu), and the tetramerization domain (Arg226Gln, Asn247Ser, and Glu249Gln) contribute to the classic neonatal and infantile onset of developmental regression. The HSD10 protein functions through a homotetrameric complex, which catalyses mitochondrial tRNA (mt‐tRNA) maturation as well as mt‐tRNA methylation through a subcomplex with the methyltransferase subunit in the mitochondrial RNase P complex (TRMT10C) for methyltransferase activity. Vilardo et al (2015)^23^ revealed that Arg130Cys, Pro210Ser, Arg226Gln, and Asn247Ser significantly prevented mt‐tRNA from maturation. In contrast, Arg130Cys, Arg226Gln and Asn247Ser in the tetramer subunit interacting domains (subunit interaction domain and tetramerization domain) had various levels of impact to the interaction of HSD10 with TRMT10C. Therefore, *HSD17B10* pathogenic variants around tetramer subunit interacting domains contributing to the mt‐tRNA maturation and downstream function are more likely causing a more severe phenotype of disease than those variants resulting in defective HSD10 dehydrogenase function. Due to the limited number of variants evaluated in these studies, correlation of functional impact of variants in differing domains remains to be seen

Thirty females with heterozygous pathogenic variants in *HSD17B10* including index cases and relatives have been reported Table 1. Among 30 heterozygous females, approximately 13% (4/30) including our case have infantile onset HSD10 disease. All four females with HSD10 disease, including our case, present with delayed neurologic development and/or regression, which is similar to the clinical presentation seen in affected males.^12^, ^16^, ^24^, ^26^ One had a coexisting genetic diagnosis, CACNA1A‐related developmental and epileptic encephalopathy, which is most likely consistent with her neurological symptoms. Approximately 30% of heterozygous females present with an array of symptoms including learning disability, borderline intellectual disability, and dysarthria. Autism was reported in a mother of index case; however, she was also diagnosed with 3q29 microduplication syndrome. Roughly, 43% of heterozygous female were reported as asymptomatic.^16^, ^22^, ^26^, ^32^ Phenotype of 2 heterozygous females (6.7%) were not described. Skewed XCI or non‐random XCI is generally known to play a role in the clinical variability of X‐linked disorders in female carriers. Moderately skewed and highly skewed XCI are defined as having an XCI ratios of 80:20 and >90:10, respectively.^34^ These ratios have been described in many studies.^35^ However, it has not been proven whether *HSD17B10* is affected by XCI. Correlation with a skewed XCI pattern was noted in a previous study and our case.^13^ A skewed X‐inactivation pattern (80:20) was observed in cultured fibroblasts from a severely affected female with HSD10 disease, and a random X‐inactivation pattern was observed in a female with a mild phenotype.^13^ Our proband has an XCI ratio of 77:23, and while this is not clinically skewed by definition, it is worth noting that this ratio is skewed to an extent where it is closer to moderate (80:20) than it is to random XCI, 50:50. This XCI pattern and variant, Arg147Cys which is located around the subunit interaction domain, may be contributing to the phenotypic severity in this case. XCI study of additional tissues was not done; however, patterns of XCI are typically consistent across blood and tissues in young female populations.^36^, ^37^ Therefore, the XCI ratio in blood likely represents the XCI ratio in other tissues. The mother, who has a history of mild learning disability, showed an XCI ratio of 68:32, which is considered not clinically significant. The observations in this study support the role of the XCI pattern in the clinical variability of HSD10 disease.

**TABLE 1:** Genotypes and phenotypes summary of females with pathogenic/likely pathogenic variant in *HSD17B10* gene

	Case	Genotype	Phenotype	Brain MRI
Our report	Index case (45 months)	c.439C>T p.Arg147Cys	Infantile onset Developmental delay Regression Abnormal brain MRI	Lesions at basal ganglia
	Mother of index case (32 y)	c.439C>T p.Arg147Cys	Mild LD	NA
Ensenauer et al., 2002^24^	Patient 1	c.388C>T p.Arg130Cys	Infantile onset Psychomotor and speech delay	Mild frontoparietal cortical atrophy
Poll‐The et al., 2004^22^	Mother of index case	c.364C>G p.Leu122Val	Asymptomatic	NA
Perez‐Cerda et al., 2005^12^	Patient 1 (10y)	c.740A>G p.Asn247Ser	Infantile onset Psychomotor delay Ataxic gait SNHL	Normal (at age 29 months)
	Mother of patient 1	p.Asn247Ser	Asymptomatic	NA
	Mother of patient 3	c.388C>T p.Arg130Cys	Borderline LD	NA
Cazorla et al., 2007^25^	Mother of index	c.388C>T p.Arg130Cys	Mild ID	NA
Lenski et al., 2007^32^	Mother of index case	c.574C>A p.Arg192Arg	Asymptomatic	NA
	GM of index case	c.574C>A p.Arg192Arg	Asymptomatic	NA
	Maternal great GM of index case	c.574C>A p.Arg192Arg	Asymptomatic	NA
	Maternal aunt of index case	c.574C>A p.Arg192Arg	Asymptomatic	NA
García‐Villoria et al., 2009^15^	Mother of patient 3	c.628C>T p.Pro210Ser	Mild ID	NA
	Maternal grandmother of patient 3	c.628C>T p.Pro210Ser	Mild ID	NA
	Mother of patient 4	c.388C>T p.Arg130Cys	Borderline LD	NA
	Maternal aunt of patient 4	c.388C>T p.Arg130Cys	Borderline LD	NA
	Mother of patient 6	c.628C>T p.Pro210Ser	Borderline LD	NA
Seaver et al., 2011^38^	Mother of index case	c.194T>C p.Val65Ala	Asymptomatic	NA
Zschocke et al., 2012^14^	Mother of patient 2	c.388C>T p.Arg130Cys	Asymptomatic	NA
Fukao et al., 2014^18^	Mother of index	c.460C>A p.Ala154Thr	Not described	NA
Richardson et al., 2015^33^	Mother of index case	c.194T>C p.Val65Ala	LD Autism Chromosome 3q29 microduplication syndrome	NA
Akagawa et al., 2016^17^	Mother of male siblings	c.460C>A p.Ala157Val	Not described	NA
Oerum et al., 2017^26^	Mother of patient 1	c.526G>A p.Val176Met	Asymptomatic	NA
	Patient 2	c.364C>G p.Val12Leu	Infantile onset Hypotonia Cerebella ataxia Developmental delay Dilated cardiomyopathy	NA
Waters et al., 2019^16^	Mother of index, Family 1 (39 y)	c.364C>G p.Leu122Val	Asymptomatic	NA
	Mother of index, Family 2 (32 y)	c.364C>G p.Leu122Val	Asymptomatic	NA
	Index, Family 3 (33 months)	c.364C>G p.Leu122Val	Neonatal onset Developmental delay Hypotonia Axial hypotonia Ataxia Pathogenic variant in *CACNA1A*	Normal (at age 23 months)
	Mother of index, Family 3 (21 y)	c.364C>G p.Leu122Val	Mild intellectual deficiency Speech difficulty	NA
	Mother of index, Family 4 (31 y)	c.364C>G p.Leu122Val	Asymptomatic	NA
	Maternal GM of index, Family 4 (59 y)	c.364C>G p.Leu122Val	Asymptomatic	NA

Abbreviations: GM, grandmother; ID, intellectual disability; LD, learning disability; NA, not applicable; SNHL, sensorineural hearing loss.

We report a female patient with HSD10 disease. The XCI pattern in this case supports the possibility of X‐inactivation playing a role in HSD10 disease in females. It still remains to be seen if the XCI pattern is closely correlated with the phenotypic variability among reported females and/or if there are other factors underpinning the wide array of severity observed among both males and females. Thus far, research into whether the *HSD17B10* gene escapes XCI is inconclusive. There are also several reports of mildly affected or apparently asymptomatic males in families with the same pathogenic variants as severely affected male probands who are not carrying the Leu122Val attenuated variant. Functional studies of the known causative variants in general and further analysis of XCI patterns among females are warranted to further characterise disease mechanisms and to provide insight into the observed variability among both males and females carrying pathogenic variants in the *HSD17B10* gene.

## CONFLICT OF INTEREST

The authors declare no conflict of interest.

## AUTHOR CONTRIBUTIONS

**Jariya Upadia**: planning, conducting and reporting the work described in the article. **Nicolette Walano:** reporting, revising the work described in the article. **Grace S. Noh**: reporting, revising the work described in the article. **Jiao Liu**: reporting, revising the work described in the article. **Yuwen Li**: reporting, revising the work described in the article. **Stephen Deputy**: reporting, revising the work described in the article. **Lindsay T. Elliott**: reporting, revising the work described in the article. **Joaquin Wong**: revising the work described in the article. **Jennifer A. Lee**: reporting, revising the work described in the article. **Raymond C. Caylor**: reporting, revising the work described in the article. **Hans C. Andersson**: reporting, revising the work described in the article.

## ETHICS STATEMENT

No ethical approval was required.

## INFORMED CONSENT

All procedures followed were in accordance with the Helsinki Declaration of 1975, as revised in 2000. Informed consent was obtained from the family for being included in the study.

## ANIMAL RIGHTS

This article does not contain any studies with animal subjects performed by the any of the authors.

## Data Availability

My manuscript has no associated data.
